# Factors in nephrologists’ decision to treat pre-dialysis CKD patients with vitamin D insufficiency and SHPT: A discrete choice experiment

**DOI:** 10.1371/journal.pone.0283531

**Published:** 2023-03-29

**Authors:** Anand C. Reddy, Andy Nguyen, November H. McGarvey, Matthew D. Gitlin, Charles W. Bishop, Akhtar Ashfaq

**Affiliations:** 1 Permian Basin Kidney Center, Odessa, TX, United States of America; 2 BluePath Solutions, Los Angeles, CA, United States of America; 3 OPKO Health, Inc., Miami, FL, United States of America; Istanbul University-Cerrahpasa, Cerrahpasa Medical Faculty, TURKEY

## Abstract

Little is known about the most important factors that inform a nephrologist’s decision to treat (DTT) pre-dialysis chronic kidney disease (CKD) patients with vitamin D insufficiency (VDI) and secondary hyperparathyroidism (SHPT). The objective of this study was to identify such factors and their relative importance in the DTT with a vitamin D therapy. A web-based, adaptive design conjoint analysis discrete-choice survey was developed to study factors that informed the DTT among a sample of 200 nephrologists located throughout the United States. Based on literature review and clinician input, eight attributes were selected that could influence a provider’s DTT: age, race, CKD stage, serum 25-hydroxyvitamin D (25D), parathyroid hormone (PTH), serum calcium (Ca), serum phosphorus (P), and history of comorbidities. Respondents were asked to select one patient profile most suitable for treatment from three profiles with varying attribute levels. Each attribute’s relative importance score was computed using hierarchical-Bayesian statistics to measure the influence of each factor where higher scores represented greater DTT consideration. The pooled analysis revealed the four most important factors: serum 25D (31.4%), serum Ca (22.7%), plasma PTH (11.5%) levels, and history of comorbidities (8.5%). Age (8.2%), serum P (7.7%), CKD stage (5.7%), and race (4.4%) were relatively less important. Patients’ 25D and Ca levels contributed to more than half of nephrologists’ DTT, with the consideration of PTH levels being less of a factor. Further understanding of the driving forces behind the factors that inform the DTT may help to standardize the management of CKD patients with SHPT and VDI and improve outcomes.

## Introduction

Abnormalities in mineral metabolism and bone histology begin early in the course of chronic kidney disease (CKD), worsening as estimated glomerular filtration rate (eGFR) declines [1]. Even minimal reductions in eGFR have been linked to increased risk of bone loss (osteoporosis), and hip fracture incidence increases as CKD progresses. Vitamin D insufficiency (VDI), defined as serum total 25-hydroxyvitamin D levels (25D) below 30 ng/mL, and secondary hyperparathyroidism (SHPT), defined as elevated parathyroid hormone (PTH) levels, are two CKD-related complications that reduce quality of life [2] and have other serious consequences; yet, their real-world treatment remains variable. Previous studies associate VDI with increased morbidity and mortality [3, 4] and SHPT, if not managed, with increased risk of mineral and bone disorder (MBD) and other medical conditions [5].

Although clinical practice guidelines for CKD-MBD have been issued by Kidney Disease Improving Global Outcomes (KDIGO) [6, 7] and Kidney Disease Outcomes Quality Initiative (K/DOQI) [8], active debate continues over appropriate treatment of pre-dialysis CKD patients with VDI and SHPT. Recent advances suggest that revisions to the guidelines are needed with regard to the appropriate target for serum 25D [9], and the best therapy to attain that target for control of elevated PTH [10, 11]. The traditional treatment paradigm for SHPT is associated with limited benefits and increased risks, and needs to be updated in order to improve patient care [10].

Nephrologists providing care for CKD patients vary in their experience, backgrounds, and preferences regarding patient care, and the current evidence and objective data points on which they relay to justify initiating treatment for VDI and SHPT is limited. Thus, the primary objectives of this discrete choice experiment (DCE) were to understand the relative importance of key patient attributes in a nephrologist’s decision to treat (DTT) a patient, and to explore how these attributes associate with the choice of therapy.

## Materials and methods

In this web-based DCE survey, adaptive choice-based conjoint analysis (ACBC) methodology and proprietary Sawtooth software were used to assess nephrologists’ preferences in the management of VDI and SHPT in patients with pre-dialysis CKD and MBD. ACBC, a type of “tradeoff analysis”, helped to clarify how a healthcare provider might make complex decisions, in this case the DTT, by balancing competing factors. For example, the DTT might depend on a patient’s CKD stage and serum 25D and plasma PTH levels. Similarly, a concern for potential iatrogenic adverse events associated with vitamin D therapies, such as hypercalcemia, hyperphosphatemia and hypercalciuria, might drive the choice of therapy. The ACBC technique conjoined two or more attributes of interest at a time (eg, a patient’s serum 25D level and long-term safety risk) that could act together and influence a nephrologist’s decision-making process. In the DCE survey, respondents viewed hypothetical situations with different levels of paired attributes and selected which treatment they would have choosen (if any) for the patient under each scenario. The ACBC used computer adaptive testing (CAT) to identify attributes that could most strongly influence physician treatment decision-making.

### Participant selection and recruitment

The first 200 nephrologists meeting study inclusion criteria were enrolled by a third-party panel provider, Dynata, over a three-month period. Only practicing board-certified United States (US) based physicians with nephrology as a specialty were selected and recruitment quotas related to age and region were implemented to help ensure a more representative sample in line with that recorded in the American Medical Association (AMA) Masterfile. An institutional review board (IRB) exemption was obtained from the Western IRB prior to the study initiation and recruitment of nephrologists.

### Survey design

Participants completed a web-based DCE survey reviewed and pilot tested by two clinical nephrologists. In the survey, nephrologists were asked to evaluate trade-offs in their individual decision-making regarding patient treatment based on the interplay of all included attributes. Additionally, respondents were asked several single item questions to help assess their behaviors, attitudes, and characteristics pertinent to the DTT.

**Fig 1:**
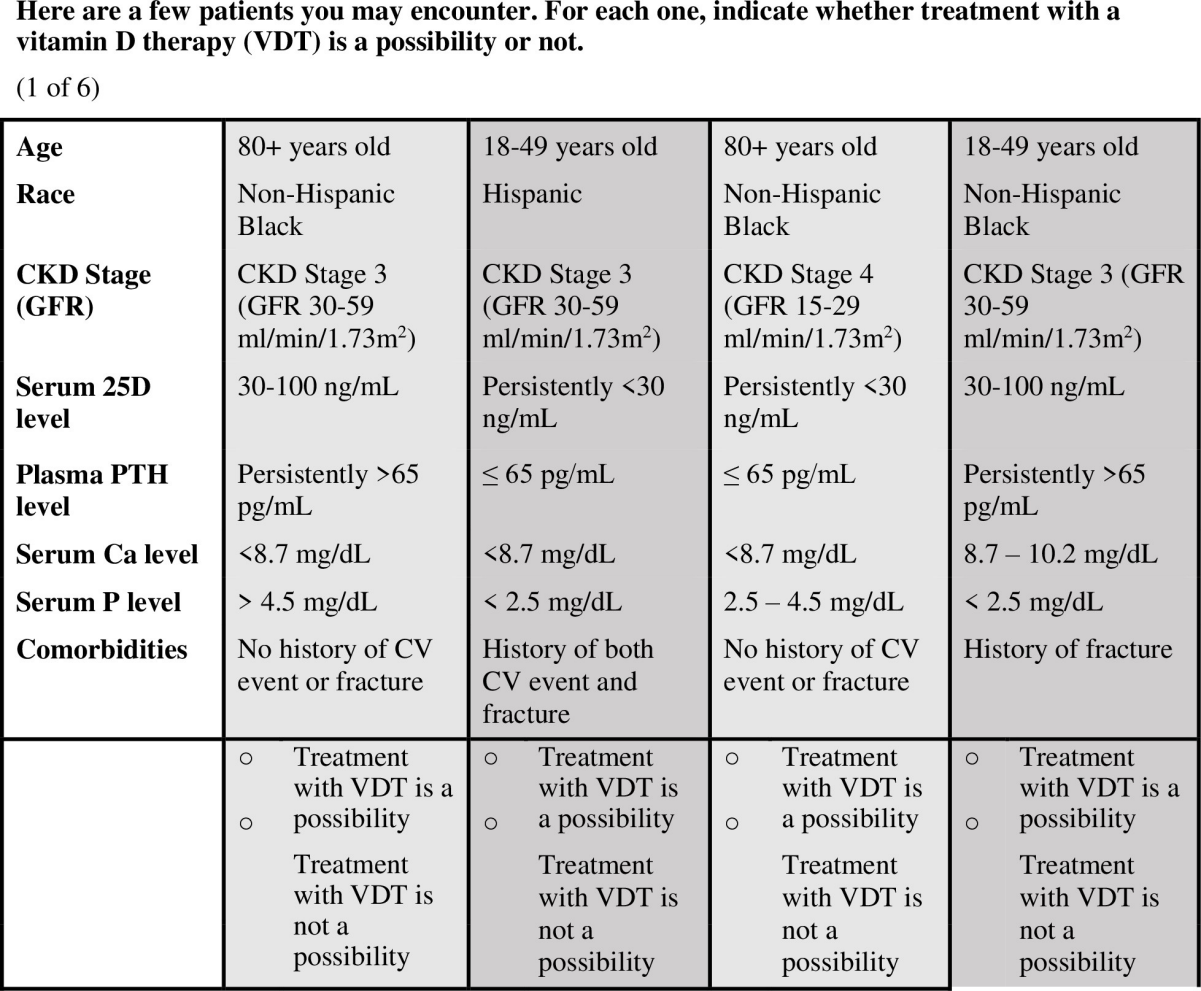
Example scenario comparison in DCE portion of the survey.

The survey was divided into three main sections. Section 1 captured provider demographics and characteristics such as age, ethnicity, race, years in practice, and facility location, urbanicity and patient volume. Further, it characterized the patient populations with regard to CKD stage and comorbidities. Section 2 asked questions probing the providers’ approaches to treating MBD, VDI and SHPT and their treatment preferences. Section 3 contained the DCE in which nephrologists were presented with hypothetical patient profiles, adapted per previous responses based on CAT programming. Hypothetical patient attributes were selected based on literature reviews and input from independent expert nephrologists. Selected patient attributes included age, race, CKD stage, comorbidities, and blood levels of 25D, PTH, calcium (Ca), and phosphorus (P) levels (Table 1). In the DCE, the respondent was first asked to select the level in each attribute indicating the situation where the management of VDI and SHPT would be most appropriate. Based on the response to this question, the respondent was shown various patient profile scenarios with varying attribute levels to determine relative importance of the attribute level in the DTT. Fig 1 shows an example of a patient profile scenario comparison with which a respondent may have been presented. Respondents were shown a total of six sets of four patient profiles. Using an adaptive and iterative process, the software determined the most crucial attributes that determined the DTT. In the process, the DCE checked for each respondent’s consistency and reliability of responses, and circled back if the respondent provided contradictory information.

**Table 1:** Attributes and levels selected for conjoint analysis.

Variable	Response Options/Levels
**Age**	18–49 years old 50–79 years old 80+ years old
**Race**	Non-Hispanic Black Hispanic Other
**CKD Stage (eGFR)**	CKD Stage 3 (eGFR 30–59 mL/min/1.73 m^2^) CKD Stage 4 (eGFR 15–29 mL/min/1.73 m^2^) CKD Stage 5 (eGFR <15 mL/min/1.73 m^2^, pre-dialysis)
**Serum 25-hydroxyvitamin D level (25D)**	Persistently vitamin D insufficient (<30 ng/mL) Vitamin D sufficient (30–100 ng/mL) High vitamin D (>100 ng/mL)
**Plasma parathyroid hormone levels (PTH)**	Normal (<65 pg/mL) High (>65 pg/mL)
**Serum Calcium (Ca) Levels**	Below normal (<8.7 mg/dL) Normal (8.7–10.2 mg/dL) Above normal (>10.2 mg/dL)
**Serum Phosphorus (P) Levels**	Below normal (<2.5 mg/dL) Normal (2.5–4.5 mg/dL) Above normal (>4.5 mg/dL)
**Comorbidities**	No history of CV event or fracture History of CV event History of fracture History of both CV event and fracture

25D, 25-hydroxyvitamin D; Ca, calcium; PTH, parathyroid hormone; P, phosphorus; CKD, chronic kidney disease; CV, cardiovascular; eGFR, estimated glomerular filtration rate.

### Data analyses

Sociodemographic, attitudinal, and clinical study variables assessed in the DCE were analyzed descriptively with means, medians, ranges, and interquartile ranges for ratings, rankings, and percentages summarizing responses to the behavioral or attitudinal questions. A hierarchical Bayesian approach and proprietary Sawtooth software was used to analyze participants’ responses to the conjoint scenarios and identify the relative importance of each attribute level, the preference weights (part-worth utilities) for each attribute and level and the degree to which respondents were willing to trade amongst attributes and levels.

The conjoint analysis was conducted in stages. The internal validity of the findings was examined first to define the final analytical cohort and to calculate preference weights to derive any estimates. Subsequently, regression analysis was conducted at the aggregate level to estimate preference parameters from the trade-off data. The initial step in the statistical analysis involved estimating “part-worth utilities,” which are index numbers that signify how much a participant values each level of each attribute. A low utility indicates less value whereas a high utility indicates more value. Part-worth utilities were estimated by comparing how a respondent’s parameter estimates differed from a larger sample drawn from the entire survey population. A Monte Carlo Markov Chain procedure was used iteratively for this comparison across 40,000 iterations, of which 20,000 were discarded. Part-worth utilities were then zero centered for ability to compare across individual and subgroups.

Subgroup analyses were conducted to evaluate possible relationships between nephrologists’ characteristics and the relative importance of specific attributes as determined by regression modeling, and t-tests across groups were used to assess significance of any differences.

## Results

Surveyed nephrologists were on average 49.4 years old and possessed a mean of 16.4 years of experience in clinical practice. The majority of their facilities were in the south (40.0%) and in urban areas (56.0%), with geographic distribution representative of the US. The estimated mean patient volume was 313.4 per month. On average (SD), 65.0% (20.7%) of the patient volume was pre-dialysis CKD patients; respondents estimated that the majority was CKD stage 3 (47.5%), followed by CKD stage 4 (33.0%), and CKD stage 5 (19.5%). The estimated top two comorbidities for CKD stage 3 were VDI (37.9%) and history of cardiovascular (CV) disease/event (27.2%); for stage 4, VDI (47.3%) and anemia (45.6%); and for stage 5, anemia (65.7%) and SHPT (64.1%) Nearly half (46.5%) of respondents reported working at a facility that did not have a CKD-MBD treatment protocol (Table 2).

**Table 2:** Nephrologists’ demographics and facility characteristics.

Nephrologist/Facility Characteristic	N = 200
**Age, mean (SD)**	49.4 (9.8)
**Years of Clinical Practice Experience, mean (SD)**	16.4 (8.7)
**Years of Experience Cohort, n (%)**	
1–10 years	66 (33.0%)
11–20 years	67 (33.5%)
21+ years	67 (33.5%)
Location of Practice, n (%)*	
West	40 (20.0%)
Northeast	40 (20.0%)
Midwest	40 (20.0%)
South	80 (40.0%)
**Urbanicity, n (%)**	
Urban	112 (56.0%)
Rural	20 (10.0%)
Suburban	68 (34.0%)
**Patient Volume (Per Month)**	
Mean (SD)	313.4 (238.9)
Minimum–Median—Maximum	10–300–3003
ND-CKD Volume, % (SD)	65.0% (20.7)
**CKD Stage (% of CKD patients)**	
CKD Stage 3	47.5%
CKD Stage 4	33.0%
CKD Stage 5	19.5%
**CKD-MBD Treatment Protocol, n (%)**	
Yes	107 (53.5%)
No	93 (46.5%)

SD, standard deviation; ND-CKD, non-dialysis chronic kidney disease.

Respondents answered questions regarding treatment intervention triggers with clinical markers, time ranges, and age considerations (S1 Table). Among CKD stages 3, 4 and 5, most respondents stated that they would not intervene with a vitamin repletion D therapy unless serum 25D levels were below 30 ng/mL (78.0%, 68.0%, and 70%, respectively); after stage 3, rates of intervention in those with normal 25D levels doubled (26.5% vs 13.0%). When asked regarding an intervention trigger for plasma PTH levels, most (48.5%) respondents selected 65–130 pg/mL in CKD stage 3. However, in CKD stages 4 and 5, the majority of respondents stated that they would not intervene until plasma PTH levels were >130 pg/mL. When asked how long they would wait until intervening for VDI or SHPT, the majority of physicians selected ≤3 months post diagnosis across all CKD stages. Lastly, a majority selected that they had no opinion regarding a patient’s age when considering whether to intervene for VDI and SHPT with a vitamin D therapy.

Respondents were asked to create a hypothetical patient profile that would be most suitable for treatment with a vitamin D therapy by selecting one level in each patient attribute (S2 Table). From the responses by the nephrologists, the ideal hypothetical patient profile was 50–77 years old (62.5%), CKD stage 4 (47.0%), vitamin D insufficiency (88.5%), persistently high plasma PTH (83.5%), normal serum Ca (48.5%), normal serum P (51.0%), and no history of CV event or fracture (34.0%).

### Conjoint analysis

**Fig 2:**
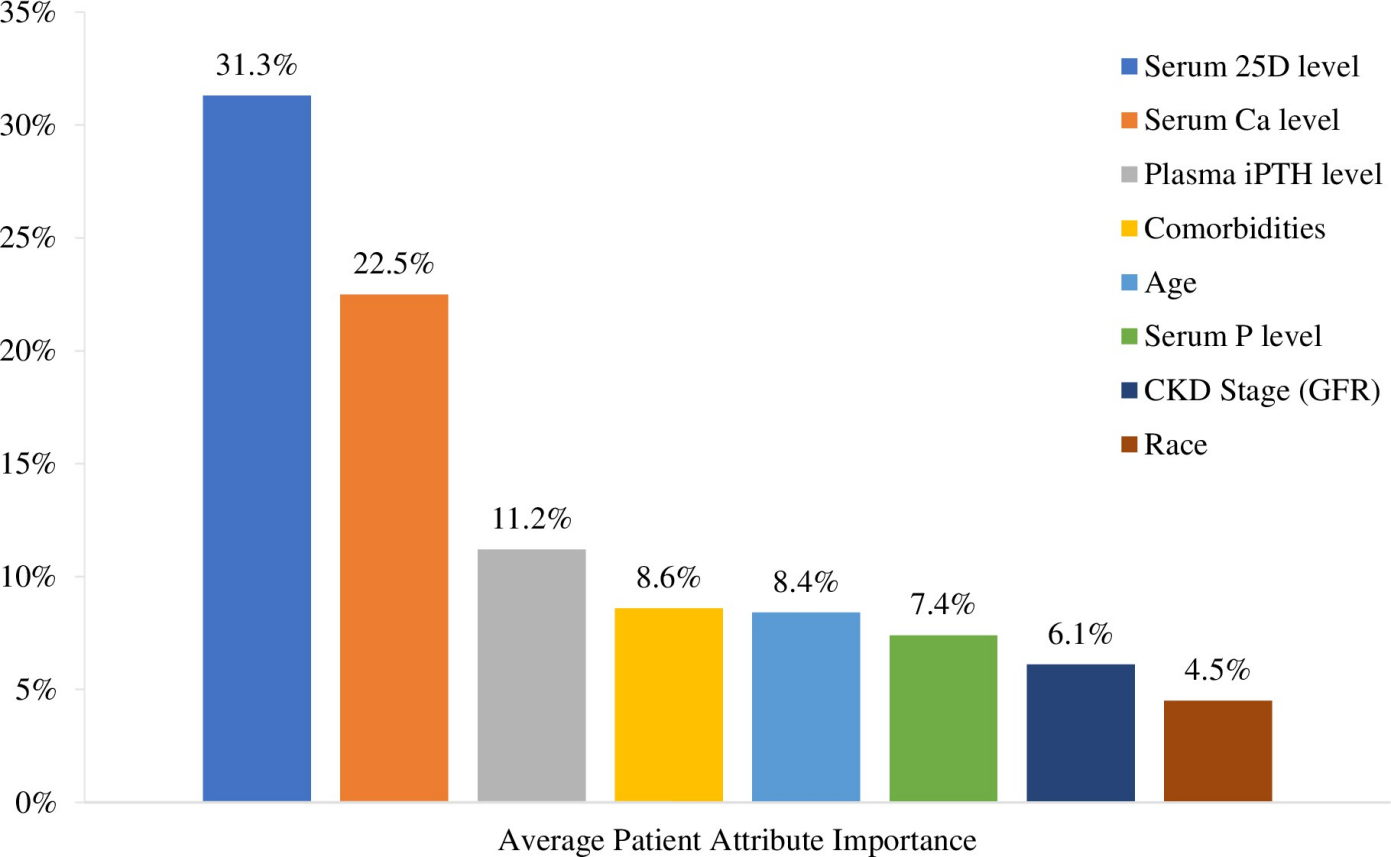
Average patient attribute importance. Factors that trigger treatment for VDI and SHPT in patients with pre-dialysis CKD stage 3–5; ordered by relative importance as indicated by conjoint analysis part-worth utilities.

The primary analysis revealed the three most important attributes in a nephrologist’s DTT were serum 25D level (31.3%), serum Ca level (22.5%), and plasma PTH level (11.2%) (Fig 2; S3 Table). Race was the least important factor (4.5%). Serum 25D was approximately three times more important than plasma PTH levels and serum Ca was on average two times more important. Respondents, on average, considered a patient’s serum Ca levels three times more than the serum P levels, with 25D ≥ 30 ng/mL if 25D <20 ng/mL at baseline.

### Part-worth utilities

**Fig 3:**
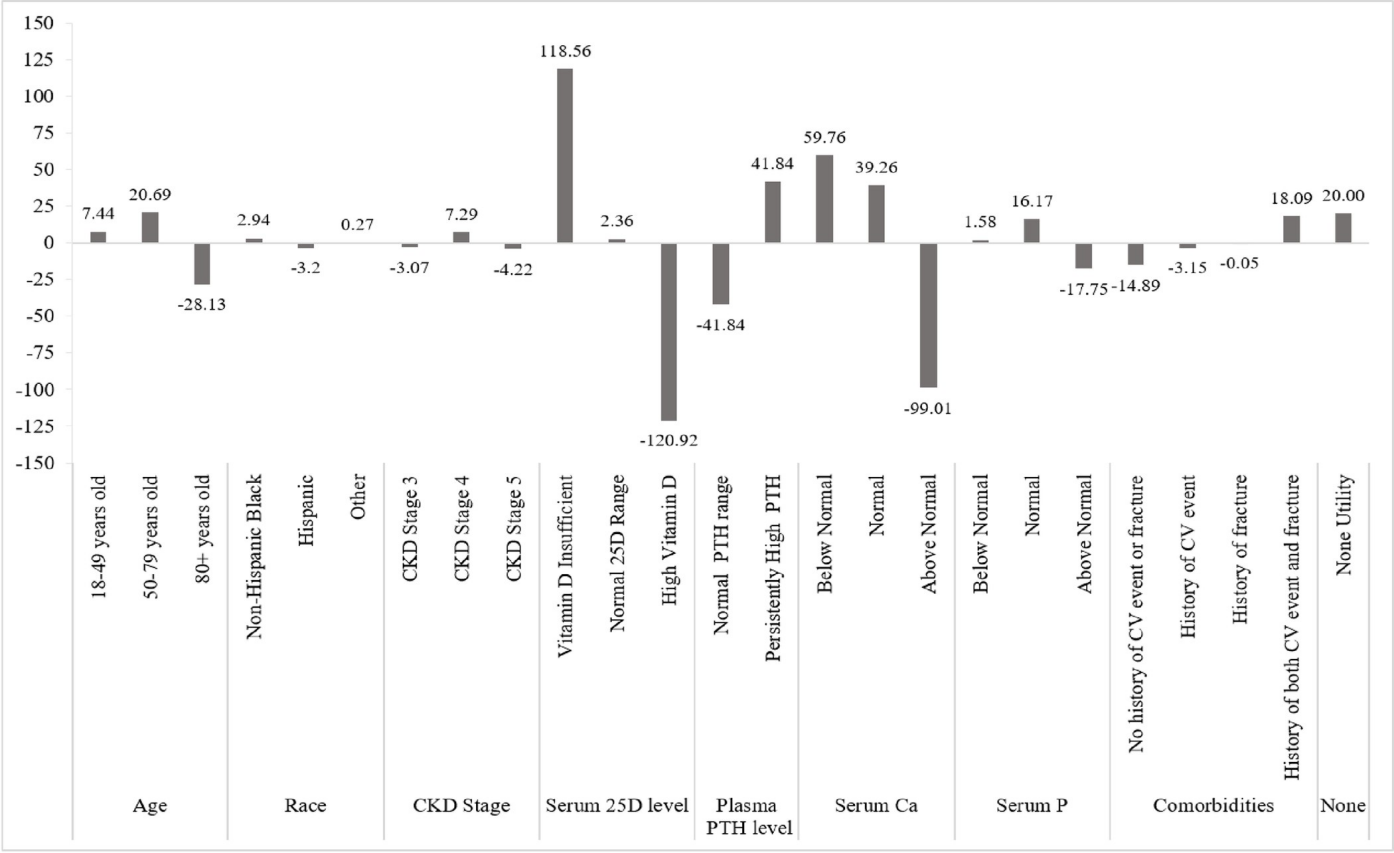
Average part-worth utilities. Displayed by patient attribute and levels within each attribute.

**Fig 4:**
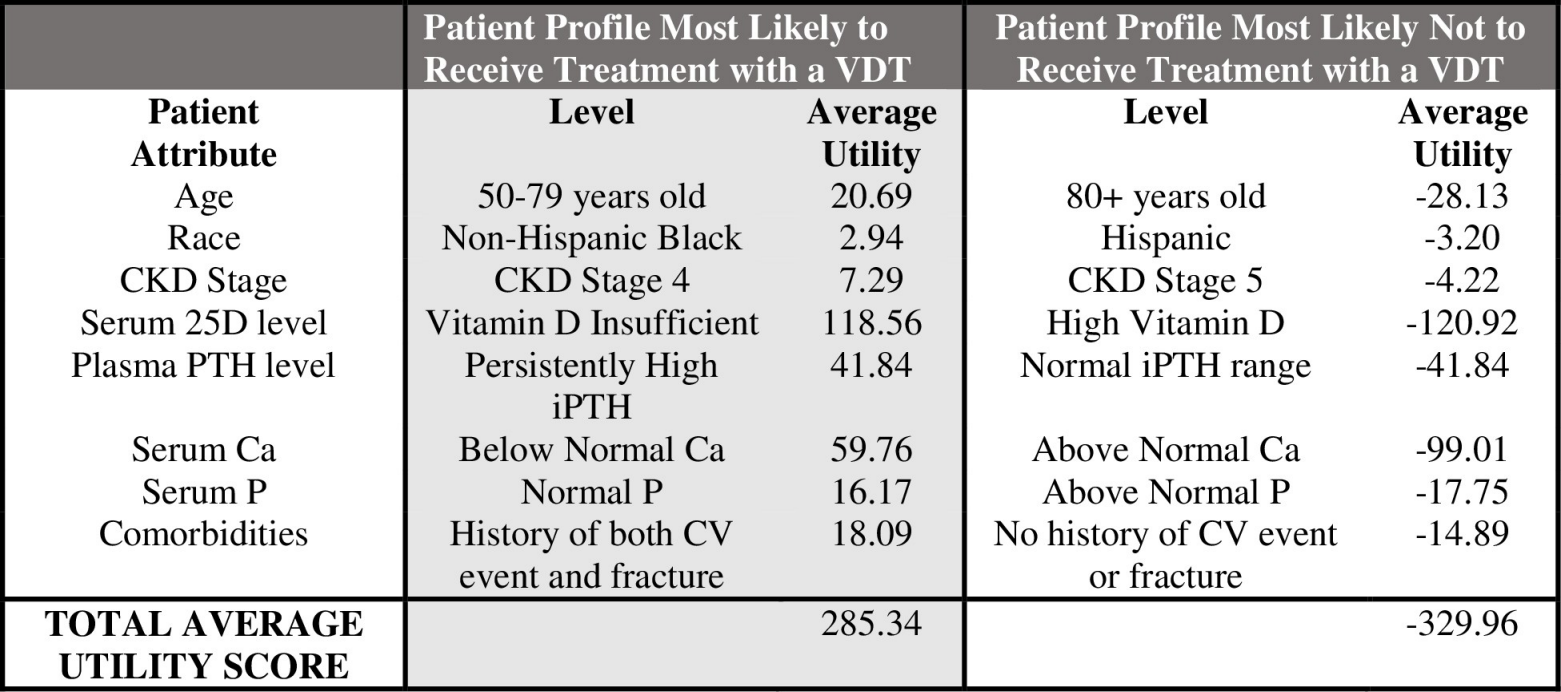
Patient profiles by likelihood to receive treatment with a VDT. Based on addition of average part worth utility values of selected patient attributes and their levels and comparison with the average part worth none utility value of 20.

Age, race, CKD stage, serum 25D, plasma PTH level, serum Ca, serum P and comorbidities were put in comparison with a “none utility” average value of 20 because apatient profile was most likely, on average, to be treated with a vitamin D therapy based on a cumulative average part-worth level utility score above 20; a score lower than 20, resulted in no treatment. Values for part-worth utilities by each attribute and level are shown in Fig 3 and S4 Table. A summary of the top and bottom levels selected for each attribute demonstrating patient profiles most and least likely to receive treatment appears in Fig 4.

Most subgroup analyses, including by nephrologist age and facility urbanicity, did not find any major significant differences in the level part-worth utility values. A subgroup analysis of whether a facility had CKD-MBD treatment protocol or not revealed statistically significant differences. When grouped by CKD-MBD treatment protocol (yes vs no), significant variation occurred in average utility value for multiple attributes: for the 18–49 years old category (4.8 vs 10.5); race, (across both subgroups, Non-Hispanic Black was the most preferred, but there were significant differences between groups in utility for Hispanic (-0.5 vs -6.3) and Other (-2.1 vs 3.0)); plasma PTH: normal (-38.2 vs -46.0) and persistently high (38.2 vs 46.0); serum Ca: below normal (48.1 vs 73.2) and above normal (-85.0 vs 115.1); serum P below normal (-2.2 vs 6.0); comorbidities: history of CV event (0.9 vs -7.9); and history of fracture (-4.6 vs 5.1).

## Discussion

The three most important attributes in a nephrologist’s decision to treat were a patient’s serum 25D level (31.3%), serum Ca level (22.5%), and plasma PTH (11.2%). A slight majority of respondents (60.0%) believed the central focus of treating SHPT was through treating VDI, consistent with the KDIGO recommendation to correct serum 25 levels that are below 30 ng/mL. Across CKD stages 3–5, the majority of physicians chose not to intervene until serum 25D reached <30 ng/mL (70–78%), regardless of persistently elevated or continuously rising PTH which KDIGO recommends treating. Serum 25D was three times more important than plasma PTH levels, while serum Ca was on average two times more important than plasma PTH in the DTT. Nearly half (46.5%) of nephrologists reported that their facilities did not have a treatment protocol for CKD-MBD.

Consistently, serum 25D and Ca levels were the two most important attributes across all subgroup analyses, while race was the least important factor in the DTT with a VDT. Most attributes were consistent across subgroups; however, some significant differences in relative attribute importance were noted between those subgroups with and without a CKD-MBD treatment protocol. Nephrologists reporting not having a protocol were slightly older (mean age: 49.9 vs 48.9 years) and possessed a little more professional experience (mean: 17.2 vs 15.8 years) compared to those with a protocol. Those nephrologists relying on their experiences rather than a treatment protocol appeared to indicate a hesitancy to treat VDI patients with normal PTH. More extensive implementation of facility treatment protocols may promote more consistent optimal treatment practices based on most recent published guidelines among CKD patients with VDI and SHPT.

All surveys may be subject to multiple sources of error, including, but not limited to sampling, coverage and measurement errors and responder bias. Notably, survey-based conjoint analyses have been validated and are thus recognized to be a valid, reliable, practical, and cost-effective method to assess preferences. Also, since only eight patient attributes were selected (in order to minimize respondent burden), combinations of attributes in the conjoint analysis did not represent all possible scenarios in the treatment of a pre-dialysis CKD patient. However, the computer adaptive methodology allowed for more variation among determinants, and content validity was assessed through review and pilot testing of the survey (and its hypothetical scenarios) by clinical nephrologists. Lastly, selection bias may have been introduced due to the nature of the purposeful sample. The nephrologists included in the study were those in the Dynata healthcare provider panel that had already indicated a willingness to complete surveys. However, respondents were largely similar in demographic characteristics to those reported in the AMA Masterfile.

## Conclusions

Patients’ serum 25D and Ca levels contributed to more than half of nephrologists’ treatment decisions, with plasma PTH a less-considered attribute. This study sheds some light into nephrologists’ therapy choices for certain patient profiles. Further exploration of the driving forces behind nephrologists’ ranking of the relative importance of these patient attributes is needed, as well as evaluation of whether these ratings of importance are in line with the current evidence and latest KDIGO guideline. Continuing such research may help to standardize management and treatment and optimize outcomes of pre-dialysis CKD patients with VDI and SHPT.

## Supporting information

S1 Table Intervention triggers. (PDF)

S2 Table Preferred patient profile suitable for treatment. (PDF)

S3 Table Relative average importance of patient attributes. (PDF)

S4 Table Average part-worth level utilities. (PDF)
