# Supplementary material for: Factors in nephrologists’ decision to treat pre-dialysis CKD patients with vitamin D insufficiency and SHPT: A discrete choice experiment
Source: PLoS One. 2023 Mar 29;18(3):e0283531. doi: 10.1371/journal.pone.0283531 (PMC10058152; doi:10.1371/journal.pone.0283531)
Supplement: S1 Table — (PDF) [file pone.0283531.s001.pdf]

S1 Table. Intervention Triggers

Question, n (%)	N = 200				
**By CKD Stage, what range of 25D would you intervene with VDT?**	**<30 ng/mL**	**30-80 ng/mL**	**80+ ng/mL**	**No opinion**	
CKD Stage 3	156 (78.0%)	26 (13.0%)	10 (5.0%)	8 (4.0%)	
CKD Stage 4	136 (68.0%)	53 (26.5%)	5 (2.5%)	6 (3.0%)	
CKD Stage 5	140 (70.0%)	42 (21.0%)	11 (5.5%)	7 (3.5%)	
**By CKD Stage, what range of iPTH would you intervene with VDT?**	**<65 pg/mL**	**65-130 pg/mL**	**>130 pg/mL**	**No opinion**	
CKD Stage 3	14 (7.0%)	97 (48.5%)	75 (37.5%)	14 (7.0%)	
CKD Stage 4	9 (4.5%)	71 (35.5%)	109 (54.5%)	11 (5.5%)	
CKD Stage 5	14 (7.0%)	46 (23.0%)	130 (65.0%)	10 (5.0%)	
**By CKD Stage, how long would you wait until intervening for VDI?**	**<3 months**	**4-6 months**	**7-12 months**	**12+ months**	**Do not intervene**
CKD Stage 3	123 (61.5%)	47 (23.5%)	15 (7.5%)	7 (3.5%)	8 (4.0%)
CKD Stage 4	136 (68.0%)	37 (18.5%)	13 (6.5%)	7 (3.5%)	7 (3.5%)
CKD Stage 5	138 (69.0%)	34 (17.0%)	13 (6.5%)	8 (4.0%)	7 (3.5%)
**By CKD Stage, how long would you wait until intervening for SHPT?**	**<3 months**	**4-6 months**	**7-12 months**	**12+ months**	**Do not intervene**
CKD Stage 3	82 (41.0%)	73 (36.5%)	22 (11.0%)	13 (6.5%)	10 (5.0%)
CKD Stage 4	91 (45.5%)	70 (35.0%)	20 (10.0%)	9 (4.5%)	10 (5.0%)
CKD Stage 5	106 (53.0%)	53 (26.5%)	24 (12.0%)	8 (4.0%)	9 (4.5%)
**By CKD Stage, at what age would you begin to be hesitant to treat with VDT?**	**20-49 y/o**	**50-79 y/o**	**80+ y/o**	**No opinion**	
CKD Stage 3	21 (10.5%)	21 (10.5%)	40 (20.0%)	118 (59.0%)	
CKD Stage 4	17 (8.5%)	27 (13.5%)	35 (17.5%)	121 (60.5%)	
CKD Stage 5	19 (9.5%)	20 (10.0%)	41 (20.5%)	120 (60.0%)	

25D, 25-hydroxy vitamin D; CKD, chronic kidney disease; VDT, vitamin D therapy; VDI, vitamin D insufficiency; SHPT, secondary hyperparathyroidism.
