# Supplementary material for: Factors in nephrologists’ decision to treat pre-dialysis CKD patients with vitamin D insufficiency and SHPT: A discrete choice experiment
Source: PLoS One. 2023 Mar 29;18(3):e0283531. doi: 10.1371/journal.pone.0283531 (PMC10058152; doi:10.1371/journal.pone.0283531)
Supplement: S2 Table — (PDF) [file pone.0283531.s002.pdf]

S2 Table. Preferred Patient Profile Suitable for Treatment

Attribute	Level	N = 200
**Age**	18-49 years old	65 (32.5%)
	50-79 years old	125 (62.5%)
	80+ years old	10 (5.0%)
**Race**	Non-Hispanic Black	81 (40.5%)
	Hispanic	37 (18.5%)
	Other	82 (41.0%)
**CKD Stage**	CKD Stage 3	70 (35.0%)
	CKD Stage 4	94 (47.0%)
	CKD Stage 5	36 (18.0%)
**Serum 25D level**	Vitamin D Insufficient	177 (88.5%)
	Normal 25D Range	17 (8.5%)
	High Vitamin D	6 (3.0%)
**Plasma PTH level**	Normal iPTH range	33 (16.5%)
	Persistently High PTH	167 (83.5%)
**Serum Ca**	Below Normal	93 (46.5%)
	Normal	97 (48.5%)
	Above Normal	10 (5.0%)
**Serum P**	Below Normal	32 (16.0%)
	Normal	102 (51.0%)
	Above Normal	66 (33.0%)
**Comorbidities**	No history of CV event or fracture	68 (34.0%)
	History of CV event	40 (20.0%)
	History of fracture	27 (13.5%)
	History of both CV event and fracture	65 (32.5%)

25D, 25-hydroxy vitamin D; Ca, calcium; iPTH, intact parathyroid hormone; P, phosphorus; CKD, chronic kidney disease; CV, cardiovascular.
