# Supplementary material for: Factors in nephrologists’ decision to treat pre-dialysis CKD patients with vitamin D insufficiency and SHPT: A discrete choice experiment
Source: PLoS One. 2023 Mar 29;18(3):e0283531. doi: 10.1371/journal.pone.0283531 (PMC10058152; doi:10.1371/journal.pone.0283531)
Supplement: S3 Table — (PDF) [file pone.0283531.s003.pdf]

**S3 Table. Relative Average Importance of Patient Attributes**

| Attribute | Average Importance | SD | Lower 95% CI | Upper 95% CI |
|---|---|---|---|---|
| Serum 25D level | 31.3% | 16.5% | 29.0% | 33.6% |
| Serum Ca level | 22.5% | 12.6% | 20.7% | 24.2% |
| Plasma PTH level | 11.2% | 6.2% | 10.3% | 12.0% |
| Comorbidities | 8.6% | 5.6% | 7.8% | 9.3% |
| Age | 8.4% | 7.0% | 7.5% | 9.4% |
| Serum P level | 7.4% | 5.0% | 6.7% | 8.1% |
| CKD Stage (GFR) | 6.1% | 4.5% | 5.4% | 6.7% |
| Race | 4.5% | 3.9% | 4.0% | 5.1% |
| **Total** | **100%** | | | |

SD, standard deviation; CI, confidence interval; 25D, 25-hydroxyvitamin D; Ca, calcium; PTH, parathyroid hormone; P, phosphorus; CKD, chronic kidney disease; GFR, glomerular filtration rate.
