# Supplementary material for: Factors in nephrologists’ decision to treat pre-dialysis CKD patients with vitamin D insufficiency and SHPT: A discrete choice experiment
Source: PLoS One. 2023 Mar 29;18(3):e0283531. doi: 10.1371/journal.pone.0283531 (PMC10058152; doi:10.1371/journal.pone.0283531)
Supplement: S4 Table — (PDF) [file pone.0283531.s004.pdf]

S4 Table. Average Part-Worth Level Utilities

Patient Attribute	Level	Average Utility	SD	Lower 95 CI	Upper 95 CI
Age	18-49 years old	7.44	7.44	7.44	7.44
	50-79 years old	20.69	20.69	20.69	20.69
	80+ years old	-28.13	-28.13	-28.13	-28.13
Race	Non-Hispanic Black	2.94	21.17	0.00	5.87
	Hispanic	-3.20	17.81	-5.67	-0.73
	Other	0.27	21.99	-2.78	3.31
CKD Stage	CKD Stage 3	-3.07	26.94	-6.80	0.66
	CKD Stage 4	7.29	22.06	4.23	10.35
	CKD Stage 5	-4.22	26.93	-7.95	-0.49
Serum 25D level	Vitamin D Insufficient	118.56	73.42	108.38	128.74
	Normal 25D Range	2.36	33.54	-2.29	7.01
	High Vitamin D	-120.92	78.03	-131.73	-110.11
Plasma PTH level	Normal iPTH range	-41.84	29.42	-45.91	-37.76
	Persistently High PTH	41.84	29.42	37.76	45.91
Serum Ca	Below Normal	59.76	53.04	52.41	67.11
	Normal	39.26	34.60	34.46	44.05
	Above Normal	-99.01	75.61	-109.49	-88.54
Serum P	Below Normal	1.58	27.81	-2.27	5.43
	Normal	16.17	19.31	13.50	18.85
	Above Normal	-17.75	33.69	-22.42	-13.08
Comorbidities	No history of CV event or fracture	-14.89	35.01	-19.74	-10.03
	History of CV event	-3.15	27.50	-6.96	0.66
	History of fracture	-0.05	22.73	-3.20	3.10
	History of both CV event and fracture	18.09	29.44	14.01	22.17
None	None Utility	20.00	120.64	3.28	36.72

SD, standard deviation; CI, confidence interval; 25D, 25-hydroxyvitamin D; Ca, calcium; PTH, parathyroid hormone; P, phosphorus; CKD, chronic kidney disease; CV, cardiovascular.
